# Species Delineation and Comparative Genomics within the *Campylobacter ureolyticus* Complex

**DOI:** 10.1128/jcm.00046-23

**Published:** 2023-04-27

**Authors:** Joel J. Maki, Mondraya Howard, Sara Connelly, Matthew A. Pettengill, Dwight J. Hardy, Andrew Cameron

**Affiliations:** a Department of Pathology and Laboratory Medicine, University of Rochester Medical Center, Rochester, New York, USA; b Department of Pathology, Anatomy, and Cell Biology, Thomas Jefferson University, Philadelphia, Pennsylvania, USA; c Department of Microbiology and Immunology, University of Rochester Medical Center, Rochester, New York, USA; Medical College of Wisconsin

**Keywords:** *Campylobacter ureolyticus*, pangenome, breast abscess, anaerobe, macrolide, *Campylobacter*, macrolides, whole-genome sequencing

## Abstract

Campylobacter ureolyticus is an emerging pathogen increasingly appreciated as a common cause of gastroenteritis and extra-intestinal infections in humans. Outside the setting of gastroenteritis, little work has been done to describe the genomic content and relatedness of the species, especially regarding clinical isolates. We reviewed the epidemiology of clinical C. ureolyticus cultured by our institution over the past 10 years. Fifty-one unique C. ureolyticus isolates were identified between January 2010 and August 2022, mostly originating from abscesses and blood cultures. To clarify the taxonomic relationships between isolates and to attribute specific genes with different clinical manifestations, we sequenced 19 available isolates from a variety of clinical specimen types and conducted a pangenomic analysis with publicly available C. ureolyticus genomes. Digital DNA:DNA hybridization suggested that these C. ureolyticus comprised a species complex of 10 species clusters (SCs) and several subspecies clusters. Although some orthologous genes or gene functions were enriched in isolates found in different SCs and clinical specimens, no association was significant. Nearly a third of the isolates possessed antimicrobial resistance genes, including the *ermA* resistance gene, potentially conferring resistance to macrolides, the treatment of choice for severe human campylobacteriosis. This work effectively doubles the number of publicly available C. ureolyticus genomes, provides further clarification of taxonomic relationships within this bacterial complex, and identifies target SCs for future analysis.

## INTRODUCTION

Campylobacter is a genus of microaerophilic Gram-negative bacteria within the phylum Campylobacterota (1–4). Campylobacter spp. are responsible for campylobacteriosis, a leading cause of zoonotic and foodborne illness worldwide (5–7). Within Campylobacter, C. jejuni and C. coli are the species most commonly associated with campylobacteriosis in humans (8). Several additional Campylobacter spp., termed the “emerging” Campylobacter spp., are acknowledged as causes of gastroenteritis and extra-intestinal infections (9). C. ureolyticus is an emerging Campylobacter spp. (10, 11).

C. ureolyticus was originally named *Bacteroides corrodens* due to colonies which pitted or “corroded” the agar surface (12). Due to its urease activity, the organism was renamed Bacteroides ureolyticus, differentiating it from facultatively anaerobic isolates of *B. corrodens* (now Eikenella corrodens) (13, 14). The organism was reclassified to the genus Campylobacter based on 16S rRNA gene sequencing (14).

C. ureolyticus may be a more common cause of bacterial gastroenteritis than previously recognized. Some reports have suggested that this species may be the second-most common cause of Campylobacter gastroenteritis after C. jejuni (15, 16). C. ureolyticus is not currently targeted by clinical molecular-based syndromic diagnostic panels, and recovery from stool culture may be complicated because it deviates greatly from the culture conditions utilized for the isolation of more well-known Campylobacter species. This suggests that the true incidence of C. ureolyticus gastroenteritis is underestimated (15, 17). Clinical case reports have also described C. ureolyticus as a cause of extra-intestinal infections, including abscesses of various tissues, necrotizing fasciitis, and uterine infections (1, 5, 18).

C. ureolyticus has been isolated in foodstuffs of bovine origin, including ground beef and unpasteurized milk products, and has been identified in the feces of cattle, swine, felines, and canines (19, 20). Unlike the summer seasonality observed for C. jejuni and C. coli, C. ureolyticus cases appear to peak in the spring, thought to coincide with increased shedding from cattle (20). C. ureolyticus has also been identified in both the human oral cavity and reproductive tract, making it difficult to attribute infections to either exogenous or endogenous sources (5, 11, 21, 22).

Beyond a few case reports and other publications, there is a paucity of research into C. ureolyticus. Here, we describe the epidemiological trends of clinical C. ureolyticus recovered in patient cultures at our institution. We used whole-genome sequencing (WGS) and pangenomics to clarify the taxonomic associations between these isolates and publicly available C. ureolyticus genomes.

## MATERIALS AND METHODS

### Clinical laboratory setting.

This study was performed under University of Rochester Medical Center (URMC) institutional review board-approved protocol no. RSRB00007269. A total of 54 C. ureolyticus (formerly Bacteroides ureolyticus) clinical isolates were identified between January 2010 and August 2022 and were cultured as part of routine clinical care in our clinical microbiology laboratory in Rochester, New York. The UR Medicine Central Laboratory serves multiple hospitals and outpatient facilities in the western New York region.

### Primary culture and identification of *Campylobacter ureolyticus* isolates.

Isolates were cultured from a variety of clinical specimen types by inoculating aerobic and anaerobic solid media, including BBL Trypticase Soy Agar with 5% sheep blood (blood agar; Becton, Dickinson and Company [BD]) and BBL Chocolate II Agar (BD) for aerobic culture, and Anaerobic Reducible Blood Agar (containing peptone, hemin, vitamin K and 5% sheep blood; Remel) or LKV Brucella agar (5% sheep blood; Remel) for anaerobic culture. Some isolates were initially recovered in thioglycolate broth (BD BBL thioglycolate broth without indicator; BD) prior to plating on solid medium. Anaerobic cultures were maintained at 35°C under an anaerobic atmosphere (5% CO_2_, 5% H_2_, 90% N_2_) in an AS-580 Anaerobe Chamber (Anaerobe Systems). Blood culture isolates were recovered on solid medium from BacT/Alert SN culture bottles (bioMérieux, Inc.) incubated on a BacT/Alert VIRTUO Microbial Detection System (or predicate device). Positive blood cultures were subcultured on anaerobic solid medium as described previously and identified using either traditional biochemical techniques (including urease testing), matrix-assisted laser desorption ionization–time-of-flight mass spectrometry (MALDI-TOF MS) using the Vitek MS (bioMérieux, Inc.) or MALDI Biotyper (Bruker), 16S rRNA gene sequencing on the MicroSeq 500 16S rDNA Bacterial Identification System (Applied Biosystems), or other phenotypic characterization methods. Of the 54 isolates, 20 had been archived at −80°C in BBL trypticase soy broth (TSB) supplemented with 20% glycerol and were available for subsequent work-up. Available isolates were subjected to further MALDI-TOF MS (without formic acid) using the MALDI Biotyper.

### Growth, maintenance, DNA extraction, and whole-genome sequencing of *Campylobacter ureolyticus*.

Archived isolates were revived and maintained on blood agar at 35°C under anaerobic conditions as described above. Growth was resuspended in 1.0 mL TSB and cells were pelleted by centrifugation at >5,000 × *g* for 10 min. Following removal of supernatant, genomic DNA was extracted using the DNeasy blood and tissue kit (Qiagen) according to the manufacturer’s instructions for Gram-negative bacteria. Extracted DNA was quantitated using the QuantiFluor dsDNA System (Promega) and a DS-11 Spectrophotometer/Fluorometer (DeNovix). Sequencing libraries were prepared using a DNA Prep Kit (Illumina) according to the manufacturer’s instructions and quantitated with the QuantiFluor dsDNA System. Paired-end sequencing was performed with a 500-cycle MiSeq reagent kit v2 on a MiSeq instrument (Illumina).

### Genome assembly, annotation, and analyses.

Paired-end reads were assembled and assessed using a CFSAN-based laboratory-developed bioinformatics pipeline as previously described (23). Briefly, adaptors and low-quality reads were removed (slidingwindow: 5:20; minlen:50) with Trimmomatic (v0.39). BowTie2 (v2.3.5.1) was used to remove reads mapping to the human genome (24, 25). Processed reads were used to generate whole-genome assemblies using SPAdes (v3.13.0) and quality was assessed with Quast (26, 27). Contigs were also uploaded to the Bacterial and Viral Bioinformatics Resource Center (BV-BRC; https://www.bv-brc.org/) to further assess assemblies for percent contamination, *N*_50_, and other assembly metrics (28). Numbers of coding sequences (CDS) and percentages of hypothetical CDSs were determined using the RAST toolkit (RASTtk) in the BV-BRC annotation portal (29).

Twenty archived C. ureolyticus isolates underwent whole-genome sequencing (WGS). Of these, one assembly (URMC_794) was contaminated with *Actinomyces* sp. and was excluded from further analysis. In addition, all publicly available genomes from the BV-BRC online genome repository (Table S1 in the supplemental material) were uploaded to the Type Strain Genome Server (TYGS) (https://tygs.dsmz.de/) to delineate taxonomic relationships to the species and subspecies levels through pairwise genomic analysis using the Genomic BLAST Distance Phylogeny approach (GBDP) with digital DNA:DNA hybridization values (dDDH, formula d4) and confidence intervals calculated through Genome-Genome Distance calculator (v3.0) (30–32). A dDDH value of >70% between two genomes indicates that those genomes belong to the same species cluster, while a dDDH value of >79% indicates that the genomes being compared belong to the same subspecies cluster (30). The TYGS tool was run in user submission mode and included C. hominis ATCC BAA-381 (GCA_000017585.1) as an outgroup. The TYGS tool infers a phylogeny from the intergenomic distances using FASTME (v2.1.6.1) (33).

Using default settings, all genomes were analyzed on Galaxy (v1.0.1, usegalaxy.eu online platform) with ABRicate (v1.0.1) and Staramr (v0.8.0), which incorporate the ResFinder, PointFinder, and PlasmidFinder databases, to identify antimicrobial resistance genes (ARGs) and plasmids (34–40). ARG regions were analyzed in BV-BRC to identify nearby mobile elements, with positional data and annotations used to generate arrow maps in R (41). The PHAge Search Tool Enhanced Release (PHASTER) tool (https://phaster.ca/) was used to predict integrated phage regions (42).

All genomes were annotated in Prokka (v1.14.6) on Galaxy to generate general feature format (gff3) files (43), which were subsequently analyzed with Roary (v3.13.0) to assess pangenomic content (44). Roary results were further fed into Scoary (v1.6.16) for statistical analysis of associations between genes and metadata (source of isolation, species, subspecies, etc.) (45). Associations between accessory genes and traits were considered significant if they possessed a Benjamini-Hochberg adjusted *P* value of <0.01 (FDR < 0.01) (46). Open reading frames (ORFs) were analyzed using eggNOG-mapper (v2.1.8) to assign Clusters of Orthologous Groups (COG) categories to genes (40, 47, 48). 16S rRNA gene sequences were identified through the BV-BRC annotation portal and aligned in Geneious Prime v2023.0.1 using Clustal Omega v1.2.2 (28, 49, 50). The Clustal Omega alignment was then used to produce an unrooted phylogenetic tree using a Jukes-Cantor distance model and a neighbor-joining methodology. Statistical analyses and visualization were conducted in R (41).

### Data availability.

The data sets presented in this study can be found in online repositories. Genomes and sequence reads are available at the NCBI Microbial Genomes repository and the NCBI Short Read Archive (BioProject ID PRJNA914250). Accession number(s) can be found in Table 1.

**TABLE 1:** Summary of genome assemblies for Campylobacter ureolyticus clinical isolates sequenced in this study^*a*^

Isolate	Paired reads (*n*)	Avg read depth (×)	Genome size (bp)	Contigs	*N*_50_	*L*_50_	CDS with functional assignments	Total CDS	Hypothetical proteins (%)	Accession no.
URMC_776	172,224	38.6	1,751,820	105	309,678	3	1,075	1,863	42.30	JAPXGZ000000000
URMC_777	302,921	63.3	1,861,891	72	150,526	4	1,095	1,951	43.87	JAPXGY000000000
URMC_778	305,505	73.1	1,653,896	16	301,919	3	1,033	1,702	39.31	JAPXGX000000000
URMC_779	257,023	54.4	1,846,058	68	316,598	3	1,125	2,008	43.97	JAPXGW000000000
URMC_780	284,034	68.0	1,628,613	20	424,254	2	1,032	1,658	37.76	JAPXGV000000000
URMC_781	255,720	55.6	1,834,070	72	426,557	2	1,087	1,947	44.17	JAPXGU000000000
URMC_782	274,650	60.5	1,765,425	71	210,745	3	1,092	1,858	41.23	JAPXGT000000000
URMC_783	227,608	49.0	1,810,854	56	304,919	3	1,078	1,932	44.20	JAPXGS000000000
URMC_784	281,337	63.1	1,810,086	53	304,919	3	1,073	1,926	44.29	JAPXGR000000000
URMC_785	156,921	34.5	1,792,575	79	124,609	6	1,085	1,923	43.58	JAPXGQ000000000
URMC_786	227,414	49.6	1,831,306	56	299,046	3	1,123	1,972	43.05	JAPXGP000000000
URMC_787	300,287	68.1	1,789,541	51	230,483	3	1,075	1,924	44.13	JAPXGO000000000
URMC_788	397,248	86.5	1,817,788	53	375,952	3	1,078	1,932	44.20	JAPXGN000000000
URMC_789	344,205	83.1	1,622,045	11	913,502	1	1,020	1,690	39.64	JAPXGM000000000
URMC_790	434,358	104.3	1,643,698	38	218,453	3	1,032	1,717	39.90	JAPXGL000000000
URMC_791	412,626	104.8	1,543,176	16	411,780	2	1,010	1,583	36.20	JAPXGK000000000
URMC_792	372,409	83.3	1,768,899	52	319,404	3	1,094	1,899	42.39	JAPXGJ000000000
URMC_793	396,161	98.2	1,561,582	14	203,266	2	1,010	1,612	37.34	JAPXGI000000000
URMC_795	269,824	55.2	1,914,577	84	157,444	4	1,156	2,102	45.00	JAPXGH000000000

a CDS, coding sequences.

## RESULTS

### Clinical significance of *Campylobacter ureolyticus* isolates.

**FIG 1:**
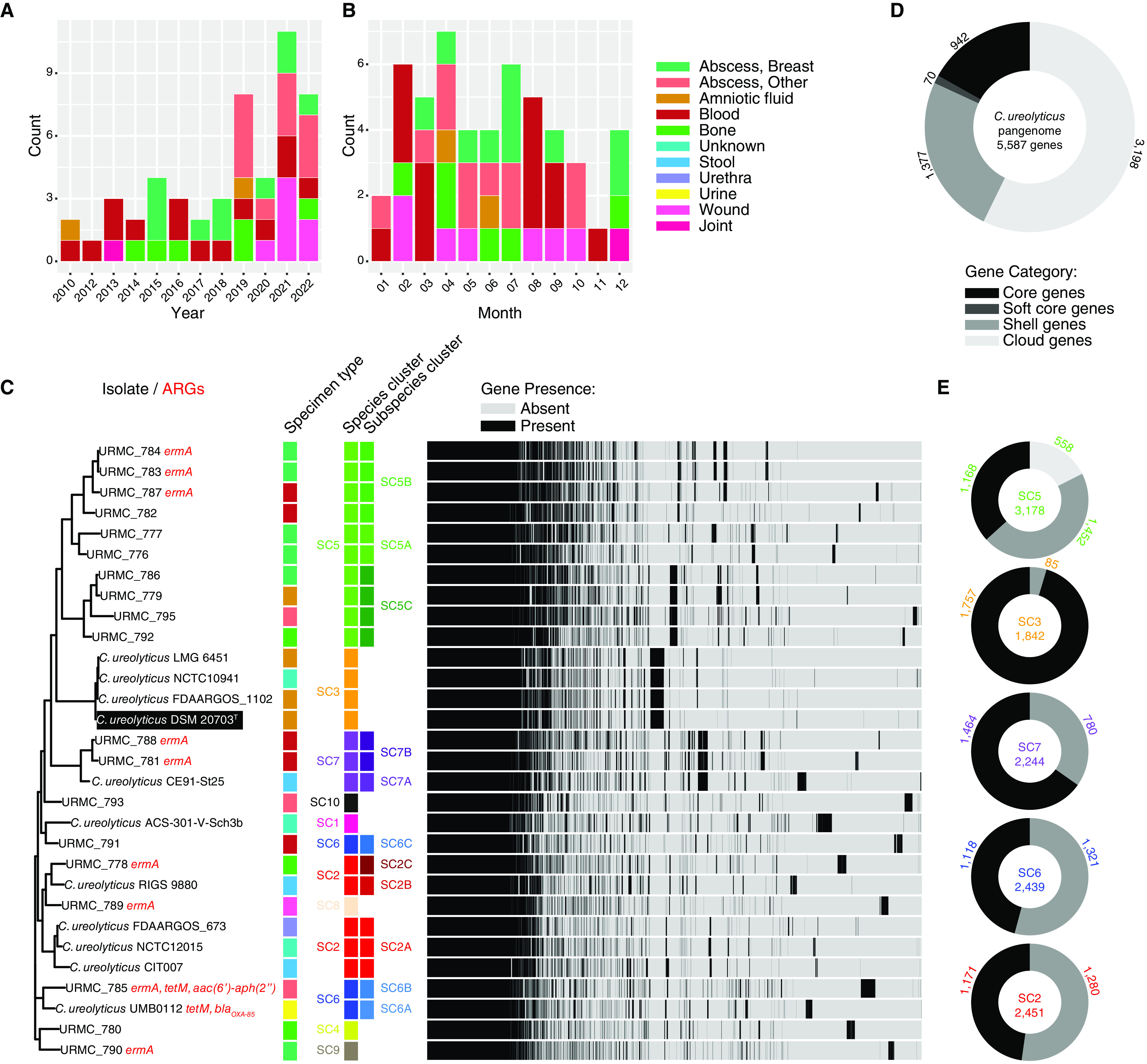
Frequency of isolation and pangenomic analysis of Campylobacter ureolyticus. Distribution of clinical isolates (*n* = 51) identified as C. ureolyticus January 2010 to August 2022. (A) Count and specimen type for C. ureolyticus isolates, by year of isolation. (B) Count and specimen type for C. ureolyticus isolates, by month of isolation. (C) Phylogenetic tree calculated from core-genome alignment of clinical isolates and publicly available genomes from the BV-BRC database, annotated with antibiotic resistance genes (ARGs), species cluster (SC), subspecies cluster, specimen type, and gene presence-absence heatmap. Scale bar represents the average number of nucleotide substitutions per position. Type strain C. ureolyticus DSM 20703^T^ indicated in black. (D) Gene pool of C. ureolyticus analyzed in this study. Genes (counts) are categorized as core (i.e., found in 99% ≤ isolates ≤ 100%), soft core (95% ≤ isolates < 99%), shell (15% ≤ isolates < 95%), cloud (0% ≤ isolates < 15%), and total genes (0% ≤ isolates ≤ 100%). (E) Pangenomic analysis of C. ureolyticus SCs composed of multiple isolates (SC2, SC3, SC5, SC6, and SC7).

A total of 54 isolates from 51 unique patients identified as Bacteroides ureolyticus in 2010, or as Campylobacter ureolyticus after taxonomic revision in 2010, were recovered between January 2010 and August 2022 (Fig. 1A) (14). C. ureolyticus was most commonly encountered in various abscesses (21/51, 41.1%); of these, 10 isolates (47.6%) were recovered from breast abscesses. Blood was the next most common specimen source (14/51, 27.5%), with all C. ureolyticus cultured from anaerobic blood culture bottles. The median time to positivity was 41.1 h for monomicrobial C. ureolyticus-only blood cultures and 37.9 h for polymicrobial blood cultures. C. ureolyticus was also cultured from wounds (7/51, 13.7%), bone infections (6/51, 11.7%), amniotic fluid (2/51, 3.9%), and a joint fluid specimen (1/51, 1.9%). For non-blood specimens, the mean time for visible colony growth was 3.1 days (range: 2 to 6 days postinoculation). C. ureolyticus detection appeared to peak during the late winter to early spring: across the entire collection period, most cases occurred in April (7/51, 13.5%), with fewer cases occurring in the late fall and early winter (Fig. 1B).

The 19 C. ureolyticus isolates were cultured from 18 unique patients; 1 patient culture had produced 2 morphotypes (URMC_783 and URMC_784). The majority of cases were polymicrobial (13/18, 72.2%); the most commonly co-isolated bacteria were anaerobic Gram-positive cocci Finegoldia magna (9/13, 69.2%) or *Actinomyces* spp. (7/13, 53.8%) (Table S2). In five cases where C. ureolyticus was the sole pathogen (5/18, 27.8%), therapy included vancomycin, cephalosporins, and β-lactam/β-lactamase inhibitors (Table S2). Two of the five patients with monomicrobial C. ureolyticus (40%) died: one was treated with vancomycin and piperacillin-tazobactam and the other received vancomycin and cefepime. Of the patients who cleared their infection, one received cephalexin, vancomycin, piperacillin-tazobactam, and tobramycin over the course of their hospital stay, while the other received piperacillin-tazobactam and ertapenem. The final case of monomicrobial C. ureolyticus infection involved a case of chorioamnionitis which resolved after induction of labor.

### Genome assembly of *Campylobacter ureolyticus* isolates from clinical specimens.

High-quality genome assemblies (*N*_50_ ≥ 124,609 bp) were produced for 19 of the 20 C. ureolyticus isolates selected for WGS, with depth of coverage ranging from 34.5× to 104.8× (Table 1). Assembled genomes ranged in size from 1.54 (URMC_791) to 1.91 Mb (URMC_795). Total CDSs ranged from 1,583 (URMC_791) to 2,102 (URMC_795). The assembly of one isolate, URMC_794, was found to be contaminated with *Actinomyces* sp. and was excluded from subsequent analysis.

### Structure of *C. ureolyticus* species clusters.

Pairwise dDDH comparison of the available genomes yielded 10 unique species clusters (dDDH > 70%) and 17 different subspecies clusters (dDDH >79%) (Fig. 1C, Table S3), suggesting that C. ureolyticus comprises a species complex.

Species cluster 1 (SC1) was composed of a single isolate, C. ureolyticus ACS-301-VSch3b. SC2 included 5 isolates further divided into 3 subspecies clusters: subspecies 2A (C. ureolyticus CIT007, FDAARGOS 673, and NCTC12015), subspecies 2B (C. ureolyticus RIGS 9880), and subspecies 2C (C. ureolyticus URMC_778). SC3 represented the “type species” of C. ureolyticus because it contained C. ureolyticus DSM 20703^T^, as well as C. ureolyticus FDAARGOS 1102, LMG 6451, and NCTC10941. SC4 was composed of a single isolate, URMC_780. SC5 was composed of 10 isolates, divided into 3 subspecies clusters: subspecies 5A included URMC_776 and URMC_777; subspecies 5B included URMC_782, URMC_783, URMC_784, and URMC_787; and subspecies 5C included URMC_779, URMC_786, URMC_792, and URMC_795. SC6 was divided into 3 subspecies, each with a single isolate: subspecies 6A was C. ureolyticus UMB0112; subspecies 6B was URMC_785; and subspecies 6C was C. ureolyticus URMC_791. SC7 was composed of 3 isolates divided into 2 subspecies: subspecies 7A contained C. ureolyticus CE91-St25 and C. ureolyticus URMC_781; and subspecies 7B included C. ureolyticus URMC_788. SC8, SC9, and SC10 were each composed of a single species: URMC_789, URMC_790, and URMC_793, respectively.

### Plasmids, phages, and antibiotic resistance genes of *Campylobacter ureolyticus*.

No identifiable plasmids were detected in either the genomes sequenced from our clinical specimens or in publicly available genomes. A total of 53 putative phage sequences were identified within all genomes, with all isolates harboring at least one integrated phage fragment and some containing multiple (Table S4). Of the identified phage sequences, 50/53 (94%) were “incomplete” and the other 3/53 (6%) were “questionable,” with no “complete” phage insertions identified. Additional fragments of multiple phage species were present in multiple C. ureolyticus isolates, with the most common hits being Haemophilus phage Aaphi23 (PHAGE_Haemop_Aaphi23_NC_004827; 7/19 [36.8%]) and *Bacillus* phage G (PHAGE_Bacill_G_NC_023719; 6/19 [31.6%]).

Putative ARGs with potential roles in tetracycline, macrolide, and aminoglycoside resistance were detected (Table S3). Two putative ARGs were detected in C. ureolyticus UMB0112: *bla*_OXA-85_ and *tetM*, conferring putative resistance to β-lactams (ampicillin) and tetracyclines. C. ureolyticus URMC_785 had the greatest number of putative ARGs: *aac*(6′)*-aph*(2′′), *ermA*, and *tetM*, conferring resistance to aminoglycosides, macrolides, and tetracyclines. Multiple isolates harbored *ermA*: URMC_778, URMC_781, URMC_783, URMC_784, URMC_787, URMC_788, URMC_789, and URMC_790. The *tetM* genes in URMC_785 and C. ureolyticus UMB0112 were immediately downstream of integrase and excisionase genes associated with a Tn*916*-like transposon (Fig. S1). The URMC_785 genome also harbored a conjugation-related protein and transcriptional regulator downstream of *tetM*, features frequently found in the genetic architecture of Tn*916*, but neither of these coding regions were present in the C. ureolyticus UMB0112 genome.

### *C. ureolyticus* pangenome analysis.

A phylogeny of the core genome alignment was annotated with the associated SCs and subspecies clusters, specimen types, and corresponding gene presence-absence heatmap (Fig. 1C). The pangenome of C. ureolyticus isolates included in this analysis comprised 5,587 genes: 942/5,587 (16.9%) were considered core genes (i.e., present in 99 to 100% of isolates), 70/5,587 (1.25%) were soft core genes (i.e., present in 95 to 99% of isolates), 1,377/5,587 (24.6%) were shell genes (i.e., present in 15 to 95% of isolates), and 3,198/5,587 (57.2%) were cloud genes (i.e., present in 0 to 15% of isolates) (Fig. 1D). The core genome alignment identified clades that corresponded to SCs and subspecies clusters. Interestingly, 5/6 (83.3%) C. ureolyticus isolates from breast abscess specimens fell into SC5, and 3/4 (75%) amniotic fluid isolates were found in SC3.

No significant associations were determined between accessory genes and the specimen types from which the C. ureolyticus isolates were derived (Table S5). Where possible, significantly overrepresented genes were noted in several of the C. ureolyticus SCs and subspecies clusters, although most were without meaningful annotation (62.9 to 88.7%). The identity and number of annotated genes was determined for SC3 (50 annotated from a total of 225 significantly overrepresented accessory genes, 22.2%), SC5 (22/73, 30.1%), subspecies 5B (13/35, 37.1%), subspecies 5C (13/115, 11.3%), and SC7 (18/129, 13.9%) (Table S5).

Next, the pangenomes for SC2, SC3, SC5, SC6, and SC7 (i.e., SCs with multiple isolates) were independently analyzed to examine the differences in the number of core, soft-core, shell, and cloud genes between the C. ureolyticus SCs (Fig. 1E). SC5 had the largest pangenome (3,178 genes), while SC3 had the smallest (1,842 genes). SC3 had a small accessory genome which included only 85 genes (5% of the total pangenome). Other SCs had accessory genomes ranging from 34.8% (SC7) to 63.2% of the pangenome (SC5).

### Comparison of *C. ureolyticus* Clusters of Orthologous Groups.

**FIG 2:**
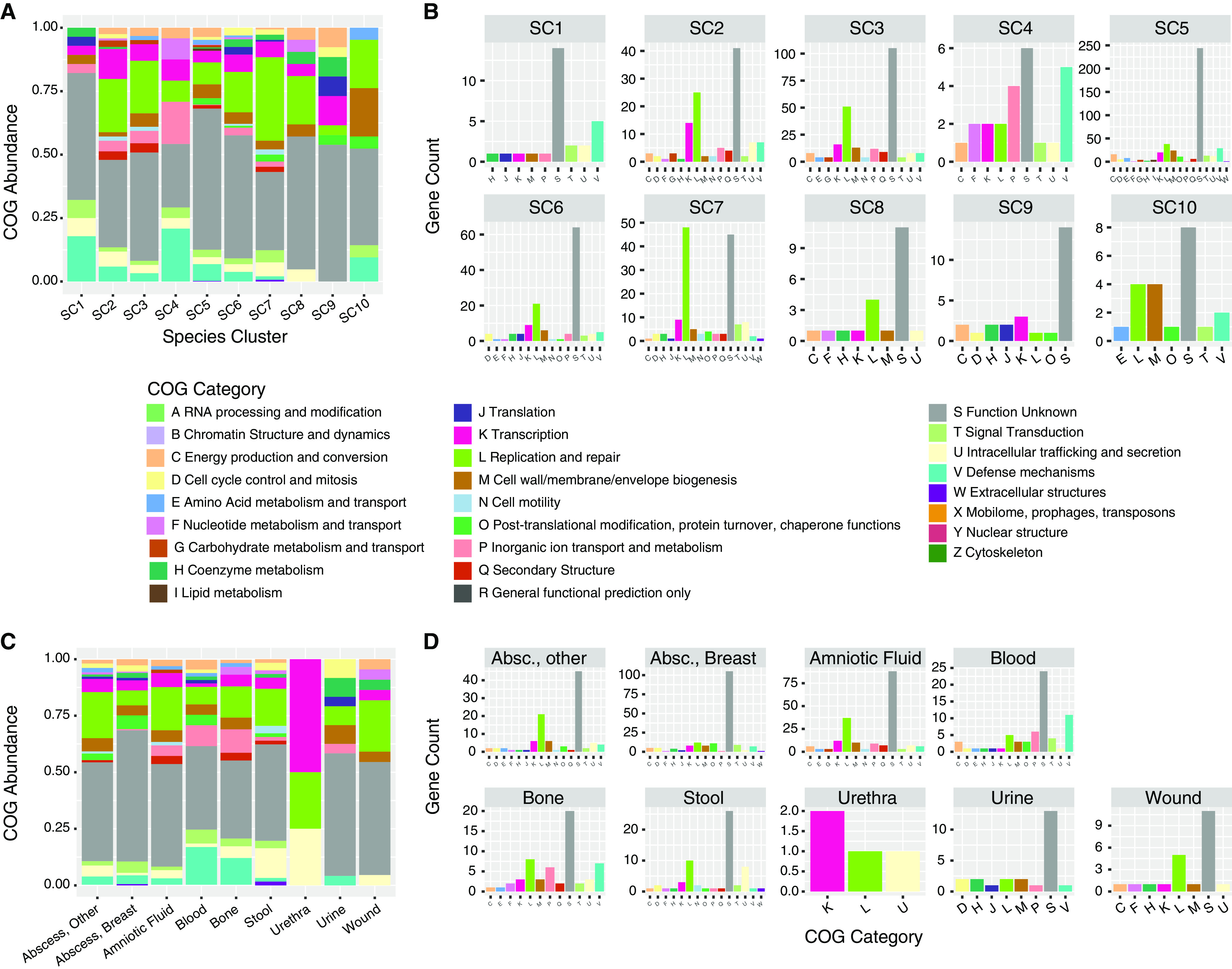
Clusters of Orthologous Groups (COG) categorization of unique genes of Campylobacter ureolyticus by SCs and specimen types. (A) Stacked graph of COG categories of unique genes for SCs. (B) COG category counts for unique genes found in different SCs. (C) Stacked graph of COG categories of unique genes for isolates from different specimen types. (D) COG category counts for unique genes found in different C. ureolyticus isolates by specimen type.

Comparison of COG categories between C. ureolyticus isolates was performed based on SCs, subspecies clusters, and specimen types, but no comparison yielded statistically significant differences (*q* < 0.05) (Fig. S2). A similar approach was used to analyze genes unique to isolates in specific SCs (Fig. 2A and B; Table S6) or specimen types (Fig. 2C and D; Table S7). While visual trends were observed, none of them were significant (*q* < 0.05). SC7 encoded the most unique genes shared between the subspecies clusters, with 38 genes unique to SC7 shared between subspecies clusters 7A and 7B (Fig. S6). SC5 had 7 unique genes conserved between subspecies clusters 5A, 5B, and 5C (Fig. S4). No unique genes were shared among the subspecies found in SC2 and SC6 (Fig. S3 and S5). The majority of genes in these analyses belonged to COG category S (“Function unknown”).

### Identification and differentiation of *C. ureolyticus* isolates by MALDI-TOF MS and 16S rRNA sequence.

Most (12/19) of the C. ureolyticus clinical isolates from our institution had been identified using the MALDI Biotyper. Isolates without an initial MALDI Biotyper score available were tested (Table S8). MALDI Biotyper scores for clinical C. ureolyticus isolates ranged between 1.70 and 2.40 (median: 1.90). Only a minority of isolates (6/19) had scored with a high-confidence identification (>2.0). No discernible trends were identified between scores and specific species and subspecies clusters (Table S8).

Full-length C. ureolyticus 16S rRNA sequences had >99.4% identity and produced a phylogenetic tree with isolates clustered by the subspecies previously determined by WGS (Fig. S7). SC3 (type species) isolates clustered together by 16S rRNA phylogeny and their 16S rRNA sequences were 100% identical. Single-nucleotide polymorphisms (SNPs) in the 16S rRNA genes were identified with the potential to differentiate some species and subspecies clusters within the C. ureolyticus complex (Table S9).

## DISCUSSION

Campylobacter spp. are a common cause of foodborne disease (6, 7), but this work shows that they are also encountered in extra-intestinal infections. Although Campylobacter is a highly diverse genus, most research and diagnostic testing has focused on C. jejuni and C. coli, such that other clinically relevant Campylobacter spp. may be underappreciated (17). C. ureolyticus counts among these, partially due to its anaerobic growth requirements (rather than microaerophilic atmospheres) and exclusion from molecular-based diagnostics platforms. When included in PCR-based screens of patients presenting with acute gastroenteritis, C. ureolyticus appears to be a common agent of Campylobacter-induced gastroenteritis, although this trend is not universal (15, 16, 51).

Although our institution does not screen for C. ureolyticus in stool, we extensively characterize bacterial isolates from patient specimens submitted for anaerobic culture. Thus, multiple isolates from extra-intestinal infections, including bone, blood, and breast abscesses, were represented in our collection. Previously, the two most common sites of C. ureolyticus we identified (abscesses and blood) had not been frequently documented in the literature: this may indicate the potential for C. ureolyticus to cause invasive disease (5, 19, 52, 53). Perhaps most interestingly, a high proportion of C. ureolyticus were isolated from breast abscesses, which had not been observed previously (52, 54, 55). Many of the patients in this study had multiple comorbidities which could have increased their susceptibility to infection, including hypertension, diabetes mellitus, and malignancy among others. However, several patients were previously healthy adults and children with no known comorbidities, making it difficult to correlate host immune status with manifestations of C. ureolyticus infection. Infections with anaerobes are frequently polymicrobial in nature (56). C. ureolyticus is no different, with the majority of isolates (72%) from this study being isolated along with other pathogens, including *Actinomyces* spp. and *Finegoldia* spp. Interestingly, both these organisms are frequently isolated from the human oral cavity, suggesting that some of the polymicrobial infections observed here may be attributed to translocation of C. ureolyticus and other bacteria from the oral mucosa to other body sites, leading to infection (57, 58). This possibility should be explored in further detail in follow-up studies.

The number of C. ureolyticus isolates increased after 2018; although there had been no change in the growth or culture techniques within our laboratory, this seems to have coincided with more frequent utilization of MALDI-TOF for the identification of anaerobes, as well as 16S PCR and sequencing, in our laboratory. Epidemiologically, isolation of C. ureolyticus peaked in April and declined in winter, somewhat matching previous epidemiological investigations of C. ureolyticus (5, 15).

Previous work suggested that there was a high degree of genomic diversity within C. ureolyticus, potentially indicating multiple genospecies (5). Here, we identified a total of 10 SCs (and several subspecies clusters) using dDDH and suggest that ‘C. ureolyticus’ is a complex of several Campylobacter species (31, 32, 59). Of these, SC3 seems to represent ‘true’ C. ureolyticus, as this cluster contained the type strain, C. ureolyticus DSM 20703^T^. SC2, SC5, SC6, and SC7 all encompassed multiple isolates and could be further classified into subspecies. SC5 represented the largest of these clusters and included most of the breast abscess isolates, which could suggest a unique association between these isolates and this clinical manifestation or pathotype. Likewise, isolates from blood fell either into SC5 or SC7, suggesting that these SCs may have greater potential for invasive infections. All SC5 isolates were cultured and sequenced at our institution, potentially indicating a unique cluster associated with our region. These do not represent a clonal outbreak because of the large SNP distance between the isolates (data not shown). While the individual associations noted here are intriguing within the context of this study, it is difficult to draw definitive conclusions between SCs and specific clinical manifestations due to the limited number of genomes available for analysis.

SC5 and SC7 were the only SCs to possess unique genes belonging to COG category W, “Extracellular structures.” Specifically, these appeared to be trimeric autotransporter adhesins (TAAs), also known as type V secretion systems (T5SSs) (60). These TAAs are primarily involved in host cell adherence/invasion, immune evasion, and biofilm formation, which could partially explain the more invasive nature of SC5 and SC7 (61–63). Additionally, SC5 and SC7 encoded 73 and 129 accessory genes, respectively, that were found to be enriched compared to other SCs within the C. ureolyticus complex. Several of these enriched genes were previously identified as secreted proteins in C. ureolyticus, including a fumarate hydratase (SC5), a metabolism-related protein, and a 3-oxoacyl-[acyl-carrier-protein] synthase (SC7), a protein involved in fatty acid metabolism (53). Several SCs, including SC7, also possessed genes for repeats-in-toxin (RTX), a pore-forming toxin protein found in the S-layer of several Gram-negative pathogens that is thought to aid in pathogenesis; it has previously been identified as a secreted protein in C. ureolyticus and other Campylobacter spp. and may be delivered to eukaryotic cells via the T5SS or another mechanism (53, 64, 65). Unfortunately, most of the significantly enriched genes were unannotated, highlighting the need for further study of the C. ureolyticus complex.

Despite the dearth of annotations, a few trends—although not significant—were observed between C. ureolyticus isolates from different specimen types. Isolates originating from blood and breast abscesses had higher percentages (4.6% and 6.0%, respectively) of unique genes associated with COG category O than isolates from other specimen types. Category O includes proteins involved in post-translational modification, protein turnover, and chaperone functions, which may play important roles in pathogen virulence and survival within the host (66, 67). Blood and bone isolates displayed higher percentages of unique genes belonging to COG category V, “Defense mechanisms,” representing 16.9% and 12.1%, respectively, of genes unique to isolates originating from these specimen types. In this category, unique genes included putative site-specific DNA-methyltransferases and various restriction-modification systems. Site-specific DNA-methyltransferases have been associated with the expression of virulence factors and regulation of host-pathogen interactions in bacterial pathogens, including Streptococcus spp. and Pseudomonas spp. (68, 69). Restriction-modification systems, which can identify and cleave foreign DNA (70), are well known in genus Campylobacter, playing roles in the regulation of virulence genes and immune evasion (71, 72). The increased cache of unique genes belonging to COG categories O and V might indicate that these “invasive” isolates of C. ureolyticus have different virulence and immune evasion properties.

No plasmids were identified in any of the C. ureolyticus complex isolates, in agreement with previous genomic analyses (73, 74). Bacteriophage elements were identified in all C. ureolyticus complex strains, although none were considered “complete.” The most complete of the integrated phages was Haemophilus phage Aaphi23 (NC_004827.1), a temperate myovirus infecting both *Aggregatibacter* spp. and Haemophilus spp. (75–77).

Relatively few ARGs were identified. The *ermA* gene, potentially conferring cross-resistance to macrolides, lincosamides, and streptogramin B, was the most abundant ARG, detected in 9/19 (47.3%) of the genomes analyzed (78, 79). Macrolide resistance in C. jejuni and C. coli is observed in 4% to 10% of clinical isolates, and the *ermA* gene has been identified in C. coli whole-genome sequences (39, 80). This enrichment of *ermA* in the C. ureolyticus complex may be concerning because macrolides, like erythromycin and azithromycin, are considered the treatment of choice for severe Campylobacter infections (most cases of Campylobacteriosis are self-limiting) (80). Otherwise, ARGs were rarely detected in the C. ureolyticus complex genomes: only 2 isolates harbored non-*ermA* resistance genes, URMC_785 and UMB0112.

URMC_785 possessed *ermA* and both *aac*(6′)*-aph*(2″) and *tetM.* The *aac*(6′)*-aph*(2″) gene may confer resistance to aminoglycosides and has been detected in other Campylobacter. Interestingly, the *aac*(6′)*-aph*(2″) in URMC_785 was flanked on one side by IS*256*, an insertion sequence often seen flanking the Tn*4001* transposable element, which frequently confers aminoglycoside resistance (81–84). The full Tn*4001* genetic architecture was incomplete in URMC_785 because it was missing a second IS*256* flanking the *aac*(6′)*-aph*(2″) gene. The *tetM* gene encodes ribosomal protection mechanisms that could impart tetracycline resistance (39, 85, 86). It is more frequently observed in Gram-positives like *Enterococcus*, but it is hypothesized that *tetO*, which is more commonly seen in Campylobacter spp., is derived from *tetM*, and a chimeric *tetM* and *tetO* hybrid was recently described in Campylobacter (87, 88). In URMC_785, *tetM* was found within a Tn*916* transposable element. Tn*916* has a broad bacterial host range and frequently hosts *tetM* (89). The other strain encoding multiple ARGs, UMB0112, encoded *tetM* (again within the Tn*916* framework) and *bla*_OXA-85_, a narrow-spectrum β-lactamase conferring resistance to ampicillin (90).

C. ureolyticus is currently a claimed (FDA-cleared) organism on the MALDI Biotyper, but it is not on the current list of cleared organisms for Vitek MS. MALDI Biotyper scores were obtained for the 19 C. ureolyticus isolates. While all isolates were at least presumptively identified as C. ureolyticus, most of those tested had scores below the cutoff (i.e., <2.00) for a high-confidence identification. Given the diversity of the isolates, additional spectra from isolates spanning the C. ureolyticus species complex may improve high-confidence identifications (91). No discernible trends were observed between MALDI Biotyper scores and either SC or subspecies cluster, although trends may emerge if more isolates from a variety of SCs are tested.

Increasingly, clinical laboratories are using 16S rRNA gene sequencing to identify bacterial isolates, particularly when MALDI and biochemical approaches give inconclusive or low-confidence results. All C. ureolyticus 16S rRNA gene sequences were >99.4% similar; however, subspecies tended to cluster together based on 16S sequence similarity. Certain SNPs in the 16S rRNA sequence were unique to some SCs and subspecies clusters (e.g., SC3 [with C44T] and subspecies cluster 5C [G1290T]); however, the usefulness of these unique sites for identifying SCs is limited due to the small number of isolates in this study.

This study has a number of other limitations. Here, the differences in pangenome content between SCs were likely impacted by disparities and the small number of genomes attributed to some SCs. Additionally, the high level of genetic similarities between the isolates found in SC3 may be reflective of resequencing efforts of the type strain, C. ureolyticus DSM 20703^T^, although this could not be confirmed through the strain metadata found on the BV-BRC database. Despite being associated with gastrointestinal disease, few of the publicly available isolates (and none of the isolates sequenced from our institution) were cultured from stool specimens. This lack of stool isolates (especially those from diarrheal disease) makes it difficult to appreciate whether C. ureolyticus extra-intestinal infections represent endogenous infections originating in the gastrointestinal track that have disseminated to the blood, bone, and other sites, or whether these infections derive from exogenous sources. Finally, although CLSI-approved standards and interpretive susceptibility testing criteria exist for C. jejuni and C. coli, none exist for C. ureolyticus. Although we did not perform susceptibility testing for all isolates, future work performed under anaerobic conditions could clarify the significance of *ermA*.

In conclusion, the analysis conducted here represents the largest performed so far for C. ureolyticus. We further explored the taxonomy of C. ureolyticus and propose that it is a complex of distinct Campylobacter species. An unexpected number of C. ureolyticus isolates, belonging to SC5, were cultured from breast abscesses and bone infections, suggesting the possibility of pathotypes within this complex. Finally, while ARGs remained relatively rare within the C. ureolyticus complex, nearly ~50% of our sequenced clinical isolates (and ~30% of all C. ureolyticus genomes) harbored the *ermA* gene. The high carriage rate of *ermA* may complicate treatment options, and the presence of ARGs associated with mobile genetic elements leads to concerns of increasing antibiotic resistance within the complex. Additional sequencing of C. ureolyticus complex isolates from other clinical specimens, animals, and food sources could allow for enhanced understanding of the genes and pathways associated with human infections and invasive disease.
