# Supplementary material for: Species Delineation and Comparative Genomics within the Campylobacter ureolyticus Complex
Source: J Clin Microbiol. 2023 Apr 27;61(5):e00046-23. doi: 10.1128/jcm.00046-23 (PMC10204631; doi:10.1128/jcm.00046-23)
Supplement: Supplemental file 1 — Supplemental material. Download jcm.00046-23-s0001.pdf, PDF file, 0.8 MB [file jcm.00046-23-s0001.pdf]

Supplementary File 1: Figures

Species Delineation and Comparative Genomics within *Campylobacter ureolyticus* Complex

Joel J. Maki[a], Mondraya Howard[a], Sara Connelly[a], Matthew A. Pettengill[b], Dwight J. Hardy[a,c], and Andrew Cameron[a]

Table of Contents:

Supplementary Figure S1. Genetic architecture of Tn*916*-like elements harboring *tetM* in *Campylobacter ureolyticus* species complex strains URMC_785 and *Campylobacter ureolyticus* UMB0112 (827.18).

Supplementary Figure S2. Stacked barcharts of Clusters of orthologous groups (COG) categories by *Campylobacter ureolyticus* complex species clusters

Supplementary Figure S3. Clusters of Orthologous Groups for genes unique to strains in *Campylobacter ureolyticus* complex species cluster 2

Supplementary Figure S4. Clusters of Orthologous Groups for genes unique to strains in *Campylobacter ureolyticus* complex species cluster 5

Supplementary Figure S5. Clusters of Orthologous Groups for genes unique to strains in *Campylobacter ureolyticus* complex species cluster 6

Supplementary Figure S6. Clusters of Orthologous Groups for genes unique to strains in *Campylobacter ureolyticus* complex species cluster 7

Supplementary Figure S7. Unrooted phylogenetic tree of 16S rRNA genes for *Campylobacter ureolyticus* genomes analyzed in this study with species cluster and subspecies cluster annotated.

Supplementary Table S1: Publicly available *Campylobacter ureolyticus* genomes from Bacterial and Viral Bioinformatics Resource Center (BVBRC) used in this study.

Supplementary Table S2: Clinical case information for clinical *Campylobacter ureolyticus* isolates sequenced in this study.

Supplementary Table S3: Type-Stain Genome Server-identified species and subspecies clusters and genome characteristics for *Campylobacter ureolyticus* genomes analyzed in this study.

Supplementary Table S4: Phage sequences identified within the *Campylobacter ureolyticus* genomes using the PHAge Search Tool – Enhanced Release (PHASTER).

Supplementary Table S5: SCOARY analysis of accessory genes associated with isolate source and *Campylobacter ureolyticus* species and subspecies clusters.

Supplementary Table S6: Unique genes belonging to individual species clusters.

Supplementary Table S7: Unique genes belonging to different isolate sources.

Supplementary Table S8: Bruker Biotyper MALDI-TOF system identity scores for *Camplyobacter ureolyticus* clinical isolates. Isolates are grouped by species and subspecies clusters.

Supplementary Table S9: Single nucleotide polymorphisms (SNPs) within the 16S rRNA gene alignment of *Campylobacter ureolyticus* genomes analyzed in this study grouped by species cluster and subspecies cluster. Unique SNPs at specific nucleotide positions as identified in the Clustal Omega alignment are highlighted in yellow and bolded, with the fraction of genomes within that species cluster or subspecies cluster noted in parentheses.

Supplementary Figures:

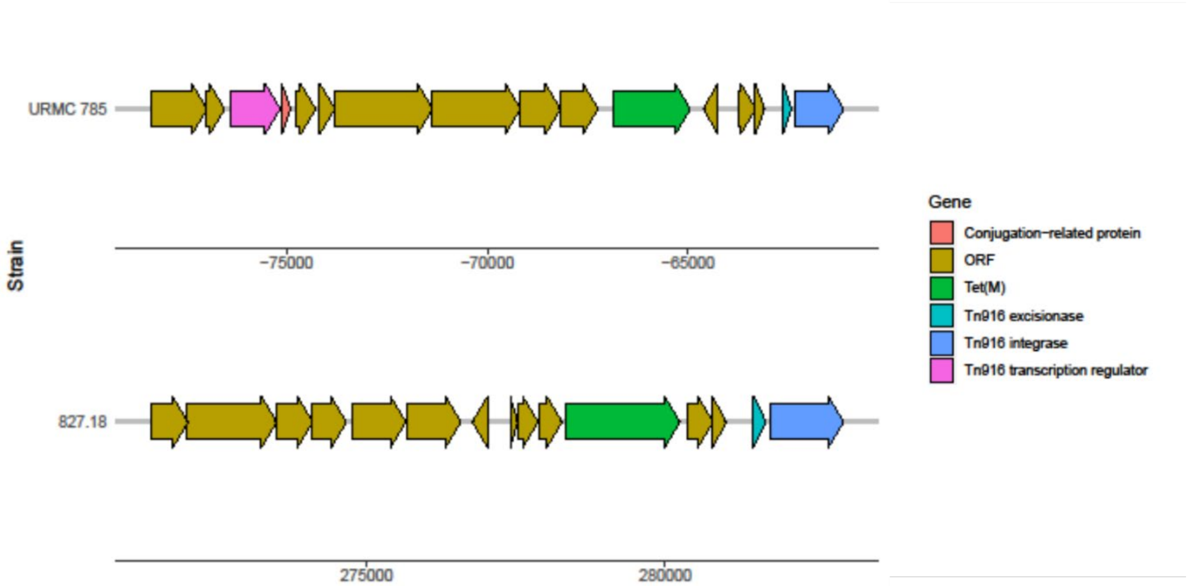

Supplementary Figure S1. Genetic architecture of Tn*916*-like elements harboring *tetM* in *Campylobacter ureolyticus* species complex strains URMC_785 and *Campylobacter ureolyticus* UMB0112 (827.18). Each arrow in the plot represents open reading frames (ORFs) as identified by the Bacterial and Viral Resource Center (BVBRC) genome annotation service, with the direction of the arrow reflection the ORF directionality and the numbers on the x-axis representing the location of the Tn*916*-like element on the contig of interest, with negative numbers representing the element being present on the reverse strand. The dark blue arrows represent the Tn*916* integrase. The teal arrows represent the Tn*916* excisionase. The green arrows represent *tetM*. The red arrow represents a conjugation-related protein. The pink arrow represents a Tn916 transcription regulator. The yellow arrows represent the other miscellaneous ORFs in the vicinity of the Tn*916*-like element.

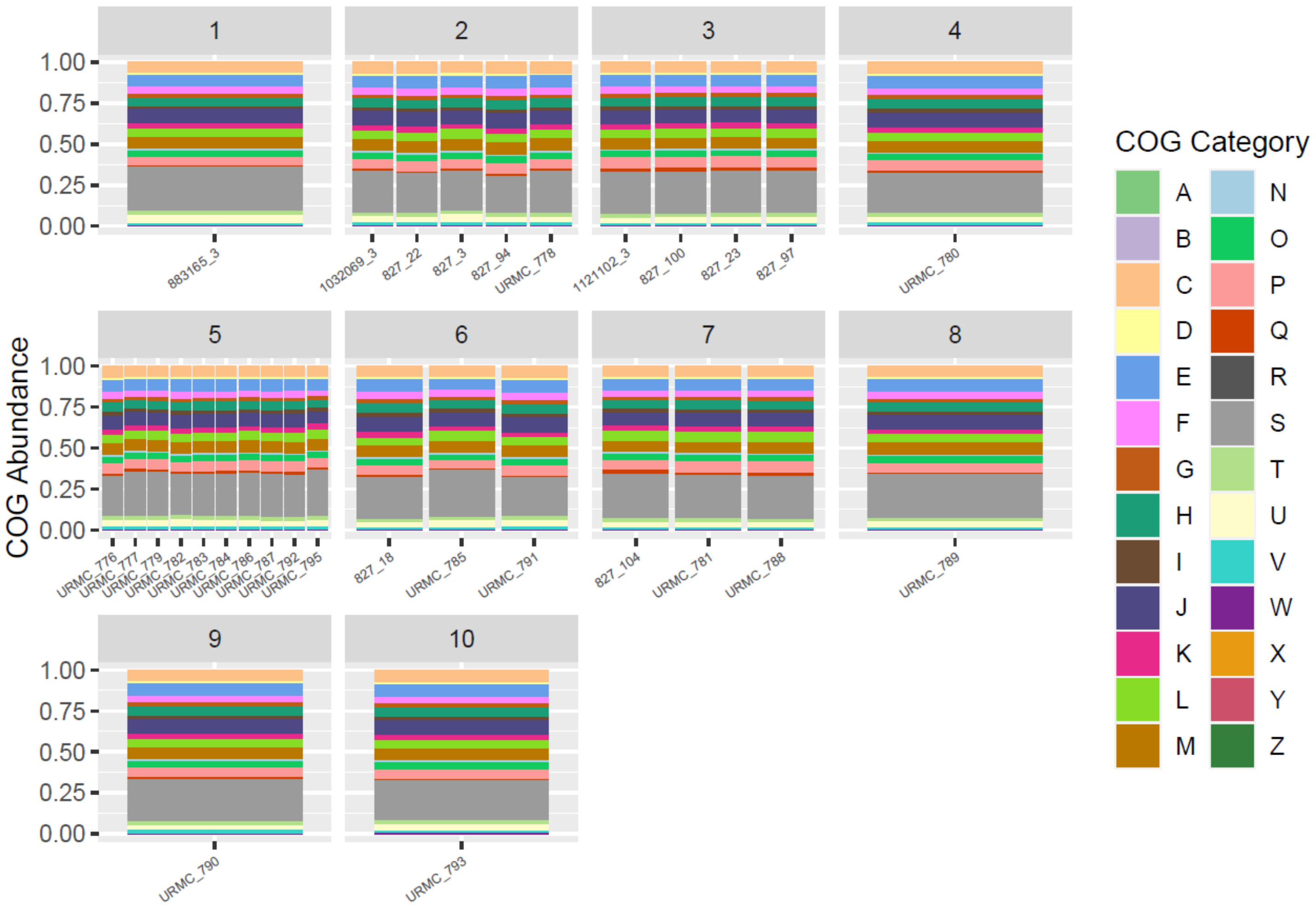

Supplementary Figure S2. Stacked barcharts of Clusters of orthologous groups (COG) categories by *Campylobacter ureolyticus* complex species clusters. Open reading frames (ORFs) were annotated with eggnog Mapper (v2.1.8) to assign COG categories to genes. Different colors represent the specific COG categories. COG categories are as follows: A: RNA processing and modification, B: Chromatin structure and dynamics, C: Energy production and conversion, D: Cell cycle control, cell division, chromosome partitioning, E: Amino acid transport and metabolism, F: Nucleotide transport and metabolism, G: Carbohydrate transport and metabolism, H: Coenzyme transport and metabolism, I: Lipid transport and metabolism, J: Translation, ribosomal structure and biogenesis, K: Transcription, L: Replication, recombination and repair, M: Cell wall/membrane/envelope biogenesis, N: Cell motility, O: Posttranslational modification, protein turnover, chaperones, P: Inorganic ion transport and metabolism, Q: Secondary metabolites biosynthesis, transport and catabolism, R: General function prediction only, S: Function unknown, T: Signal transduction mechanisms, U: Intracellular trafficking, secretion, and vesicular transport, V: Defense mechanisms, W: Extracellular structures, X: Mobilome: prophages, transposons, Y: Nuclear structure, Z: Cytoskeleton.

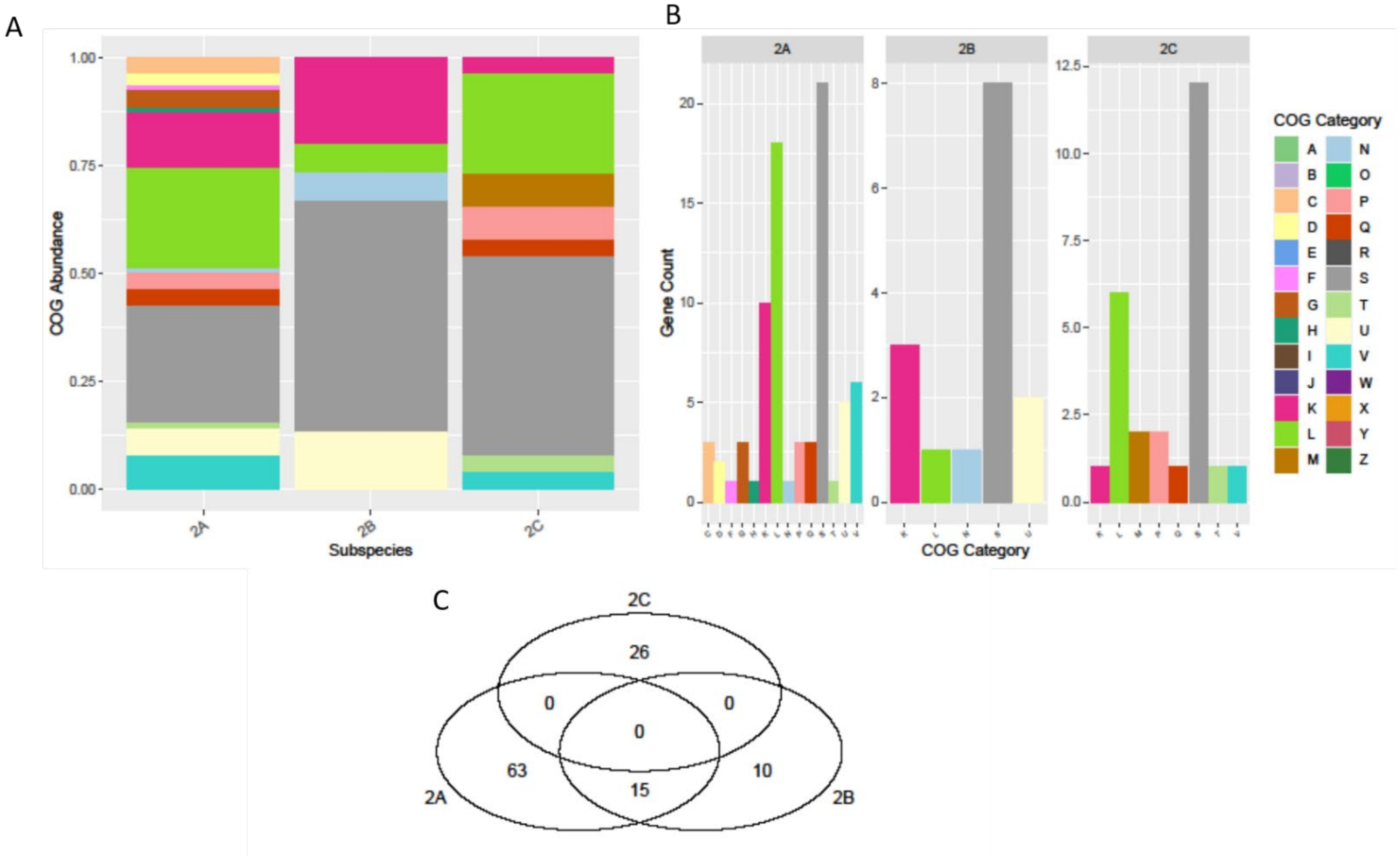

Supplementary Figure S3. Clusters of Orthologous Groups for genes unique to strains in *Campylobacter ureolyticus* complex species cluster 2 (SC2). Prokka (v1.14.6) was used to identify open reading frames (ORFs) which were annotated with eggnog Mapper (v2.1.8) to assign seed.orthologs, which were utilized to assess conserved and unique genes between different strains of *Campylobacter ureolyticus* species complex. (A) Stacked barchart of COG categories for unique genes within *Campylobacter ureolyticus* SC2, divided into subspecies clusters 2A, 2B, and 2C. (B) Barchart representing counts of unique genes within *Campylobacter ureolyticus* SC2, divided into subspecies clusters 2A, 2B, and 2C. (C) Venn Diagram of shared and unique genes between the three subspecies clusters: 2A, 2B, and 2C.

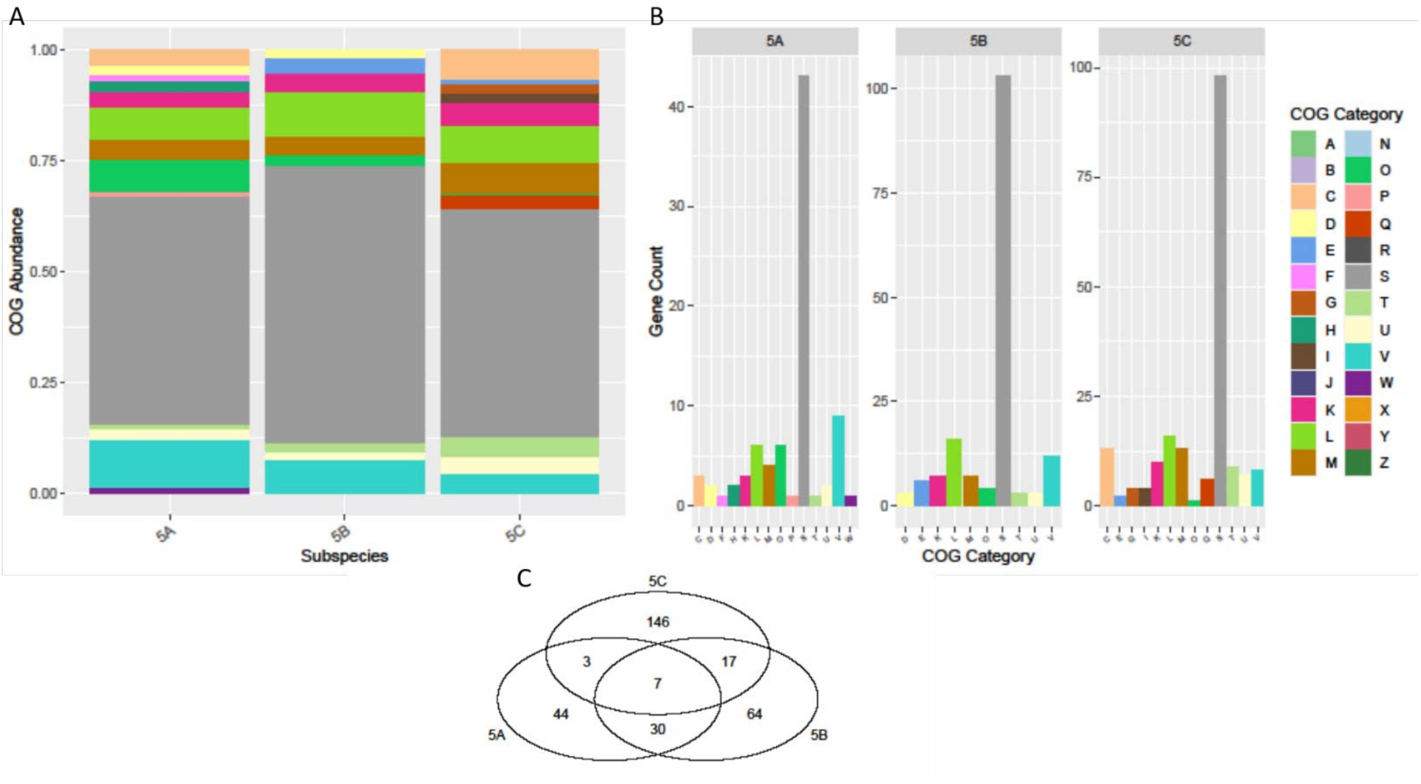

Supplementary Figure S4. Clusters of Orthologous Groups for genes unique to strains in *Campylobacter ureolyticus* complex species cluster 5 (SC5). Prokka (v1.14.6) was used to identify open reading frames (ORFs) which were annotated with eggnog Mapper (v2.1.8) to assign seed.orthologs, which were utilized to assess conserved and unique genes between different strains of *Campylobacter ureolyticus* species complex. (A) Stacked barchart of COG categories for unique genes within *Campylobacter ureolyticus* SC5, divided into subspecies clusters 5A, 5B, and 5C. (B) Barchart representing counts of unique genes within *Campylobacter ureolyticus* SC5, divided into subspecies clusters 5A, 5B, and 5C. (C) Venn Diagram of shared and unique genes between the three subspecies clusters: 5A, 5B, and 5C.

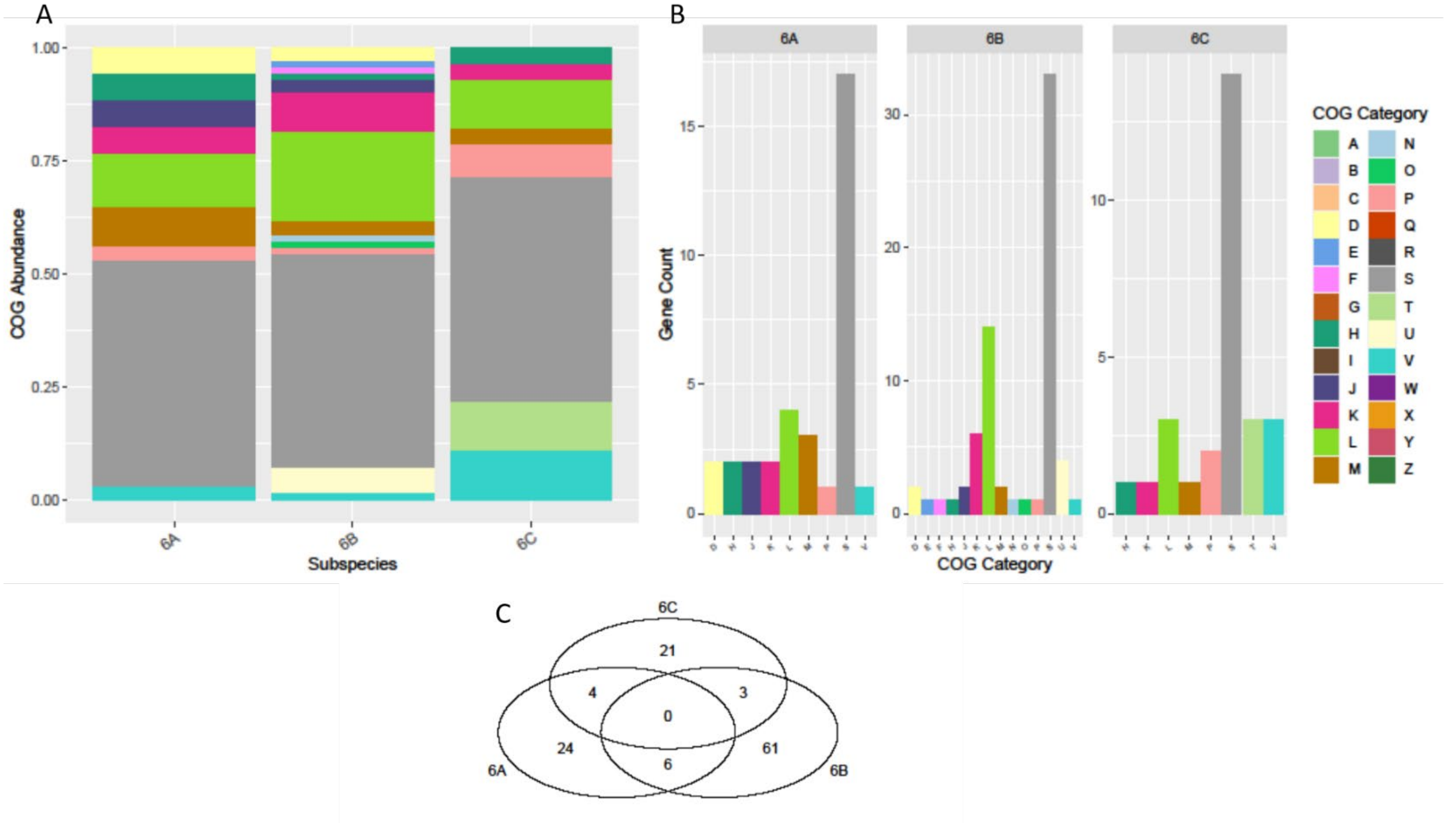

Supplementary Figure S5. Clusters of Orthologous Groups for genes unique to strains in *Campylobacter ureolyticus* complex species cluster 6 (SC6). Prokka (v1.14.6) was used to identify open reading frames (ORFs) which were annotated with eggnog Mapper (v2.1.8) to assign seed.orthologs, which were utilized to assess conserved and unique genes between different strains of *Campylobacter ureolyticus* species complex. (A) Stacked barchart of COG categories for unique genes within *Campylobacter ureolyticus* SC6, divided into subspecies clusters 6A, 6B, and 6C. (B) Barchart representing counts of unique genes within *Campylobacter ureolyticus* SC6, divided into subspecies clusters 6A, 6B, and 6C. (C) Venn Diagram of shared and unique genes between the three subspecies clusters: 6A, 6B, and 6C.

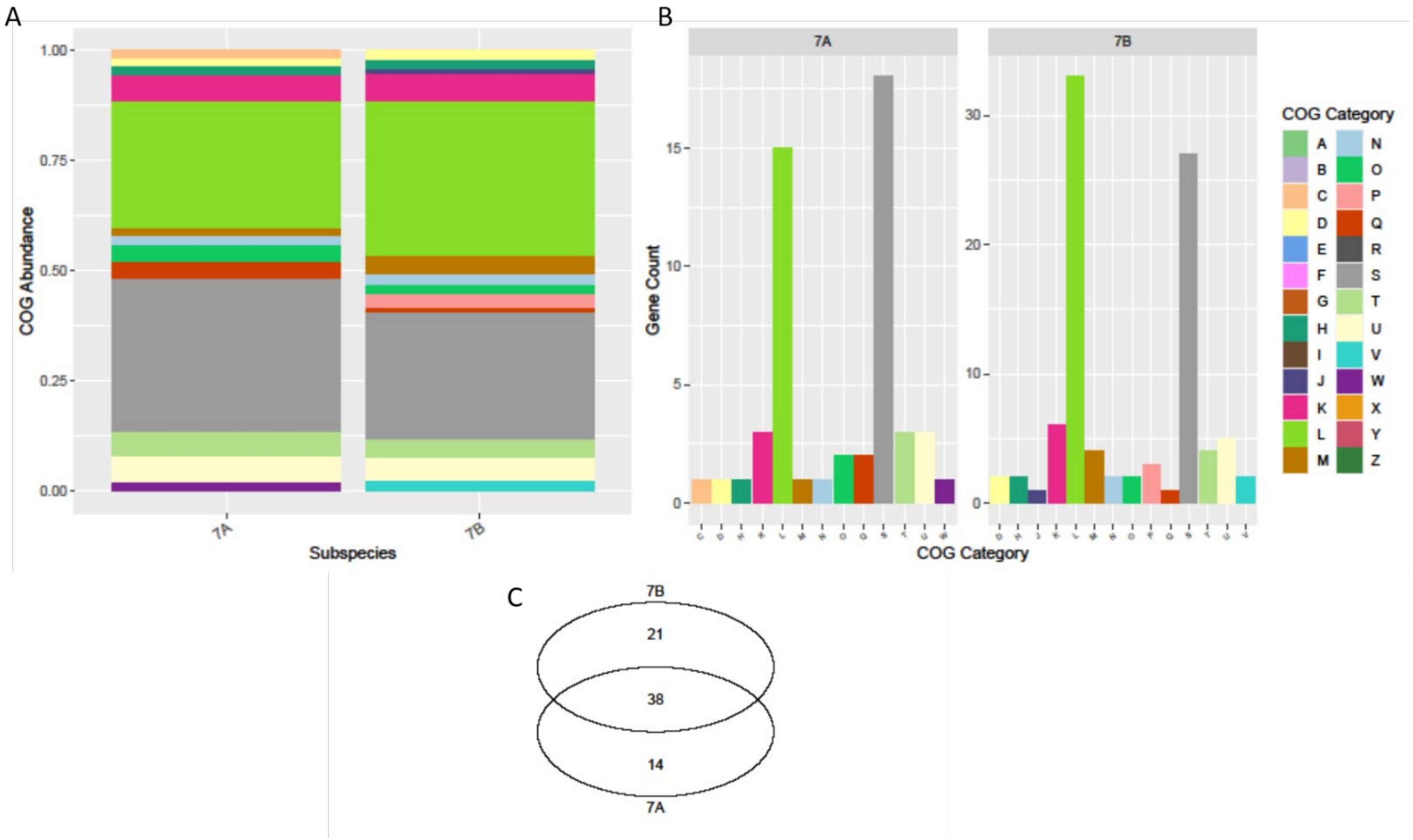

Supplementary Figure S6. Clusters of Orthologous Groups for genes unique to strains in *Campylobacter ureolyticus* complex species cluster 7 (SC7). Prokka (v1.14.6) was used to identify open reading frames (ORFs) which were annotated with eggnog Mapper (v2.1.8) to assign seed.orthologs, which were utilized to assess conserved and unique genes between different strains of *Campylobacter ureolyticus* species complex. (A) Stacked barchart of COG categories for unique genes within *Campylobacter ureolyticus* SC7, divided into subspecies clusters 7A and 7B. (B) Barchart representing counts of unique genes within *Campylobacter ureolyticus* SC7, divided into subspecies clusters 7A and 7B. (C) Venn Diagram of shared and unique genes between the three subspecies clusters: 7A and 7B.

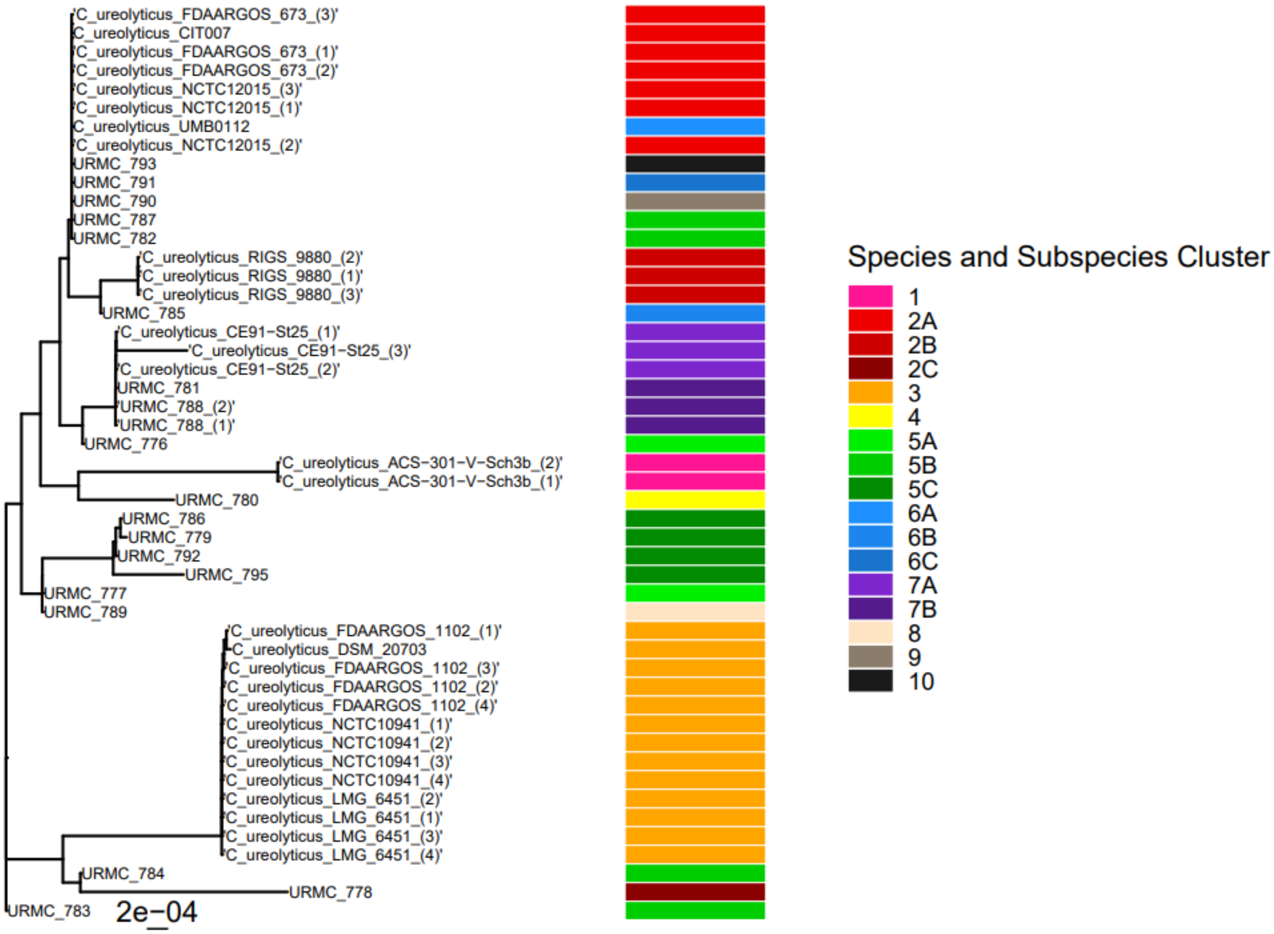

Supplementary Figure S7. Unrooted phylogenetic tree of 16S rRNA genes for *Campylobacter ureolyticus* genomes analyzed in this study with species cluster and subspecies cluster annotated. 16S rRNA genes from the same genome are differentiated with a number in parentheses. Scale bar represents the average number of nucleotide substitutions per position.

Supplementary Tables: (Tables found in Supplementary File 2)

Supplementary Table S1: Publicly available *Campylobacter ureolyticus* genomes from Bacterial and Viral Bioinformatics Resource Center (BVBRC) used in this study. Genomes were downloaded from BVBRC in August of 2022. *Denotes type strain for the species.

Supplementary Table S2: Clinical case information for clinical *Campylobacter ureolyticus* isolates sequenced in this study. Abbreviations: TPP, Time to positivity for blood cultures; DM, Diabetes mellitus; HLD, Hyperlipidemia; HTN, Hypertension; PCOS, Polycystic ovary syndrome; OSA, Obstructive sleep apnea; CHF, Congestive heart failure; RAS, Renal artery stenosis; CKD, Chronic kidney disease; COPD, Chronic obstructive pulmonary disease; DVT, Deep vein thrombosis; AFib, Atrial fibrillation; RVR, Rapid ventricular response; MI, Myocardial infarction; PAD, Peripheral arterial disease; AKA, Above-the-knee amputation; Anaerobic GPC, Anaerobic Gram-positive cocci; Pip-Tazo, Piperacillin-Tazobactam; Tri-Sulfa, Trimethoprim-sulfamethoxazole; Amox-Clav, Amoxicillin-clavulanic acid.

Supplementary Table S3: Type-Stain Genome Server-identified species and subspecies clusters and genome characteristics for *Campylobacter ureolyticus* genomes analyzed in this study. PATRIC Strain IDs are provided for the publicly available *Campylobacter ureolyticus* genomes.

Supplementary Table S4: Phage sequences identified within the *Campylobacter ureolyticus* genomes using the PHAge Search Tool – Enhanced Release (PHASTER).

Supplementary Table S5: SCOARY analysis of accessory genes associated with isolate source and *Campylobacter ureolyticus* species and subspecies clusters.

Supplementary Table S6: Unique genes belonging to individual species clusters.

Supplementary Table S7: Unique genes belonging to different isolate sources.

Supplementary Table S8: Bruker Biotyper MALDI-TOF system identity scores for *Camplyobacter ureolyticus* clinical isolates.

Supplementary Table S9: Single nucleotide polymorphisms (SNPs) within the 16S rRNA gene alignment of *Campylobacter ureolyticus* genomes analyzed in this study grouped by species cluster and subspecies cluster.
